# AntEpiSeeker: detecting epistatic interactions for case-control studies using a two-stage ant colony optimization algorithm

**DOI:** 10.1186/1756-0500-3-117

**Published:** 2010-04-28

**Authors:** Yupeng Wang, Xinyu Liu, Kelly Robbins, Romdhane Rekaya

**Affiliations:** 1 Department of Animal and Dairy Science, University of Georgia, Athens, GA 30602, USA; 2 Institute of Bioinformatics, University of Georgia, Athens, GA 30602, USA; 3 Department of Statistics, University of Georgia, Athens, GA 30602, USA

## Abstract

**Background:**

Epistatic interactions of multiple single nucleotide polymorphisms (SNPs) are now believed to affect individual susceptibility to common diseases. The detection of such interactions, however, is a challenging task in large scale association studies. Ant colony optimization (ACO) algorithms have been shown to be useful in detecting epistatic interactions.

**Findings:**

AntEpiSeeker, a new two-stage ant colony optimization algorithm, has been developed for detecting epistasis in a case-control design. Based on some practical epistatic models, AntEpiSeeker has performed very well.

**Conclusions:**

AntEpiSeeker is a powerful and efficient tool for large-scale association studies and can be downloaded from http://nce.ads.uga.edu/~romdhane/AntEpiSeeker/index.html.

## Background

Genetic association studies, which aim at detecting association between one or more genetic polymorphisms and a trait of interest such as a quantitative characteristic, discrete attribute or disease, have gained a lot of popularity in the past decade [1]. Although great progress in mapping genes responsible for Mendelian traits has been made, the genetic basis underlying many complex diseases remain unknown. It is widely accepted that these diseases may be caused by the joint effects of multiple genetic variations, which may show little effect individually but strong interactions. Such interactive effects of multiple genetic variations are often referred to as epistasis or epistatic interactions [2]. Recently, increasing numbers of studies have suggested the presence of epistatic interactions in complex diseases, e.g. breast cancer [3], type-2 diabetes [4] and atrial fibrillation [5].

A number of multi-locus approaches have been proposed to detect epistatic interactions, such as the combinatorial partitioning method (CPM) [6], restricted partitioning method (RPM) [7], the multifactor-dimensionality reduction (MDR) [3], the focused interaction testing framework (FITF) [8] and the backward genotype-trait association (BGTA) [9]. Although these methods were tested and showed promising performance on small data sets, the computational burden prohibits their application on large scale datasets.

Typically, a large scale dataset for association studies may have several tens to hundreds of thousands of markers. For example, the genome-wide case-control data set for Age-related Macular Degeneration (AMD) contains more than 100 thousand SNPs genotyped on 96 cases and 50 controls [10]. An exhaustive search of two-locus interactions needs to evaluate at least 5.00 × 10^9 ^locus combinations, and this number increases to 1.67 × 10^14 ^when three-locus interactions are considered. Although this process is computationally hard it could be enhanced by two recent approaches: the Bayesian epistasis association mapping (BEAM) [11] and SNPharvester [12], which were shown to be able to handle large scale datasets. However, more efficient and accurate methods are still desired.

The solution to this difficult search problem could be achieved using an optimization technique called ant colony optimization (ACO) algorithm. Ant colony algorithms, proposed first by Dorigio and Gambardella [13], are tools to solve difficult optimization problems such as the traveling salesman problem. ACO simulates how real ant colonies find the shortest route to a food source. Real ant colonies communicate through chemicals called pheromones, which are deposited along the path an ant travels. Ants that choose a shorter path will transverse the distance at a faster rate, resulting in more pheromones deposited along that path. Subsequent ants will then choose the path with more pheromones, thus creating a positive feedback. In ACO, artificial ants work as parallel units that communicate through a probability distribution function (PDF), which is updated by weights or pheromones. The change in pheromones is determined by some type of expert knowledge. As the PDF is updated, "paths" that perform better will be sampled at higher rates by subsequent artificial ants, and in turn deposit more pheromones. Thus, a positive feedback similar to real ant colonies is simulated.

Two recent studies showed the possibility of applying ant colony optimization to association studies [14,15]. However, the use of MDR for detecting epistatic interactions in these studies dramatically increased the computational burden. Besides, these studies did not test performance using the more practical epistatic models such as the ones proposed by Marchini et al. [16].

In this study, a new tool named AntEpiSeeker has been developed to search for epistatic interactions in large-scale association studies. The use of *χ*^2 ^values as score function to measure the association between an SNP set and the phenotype is computationally efficient. The two-stage design of ant colony optimization and the idea of searching bigger SNP sets harboring epistatic interactions enhance the power of ACO algorithms. AntEpiSeeker showed improved performance based on some practical epistatic models and large scale datasets.

## Methods

### The generic ant colony optimization

The ACO has been proven to be a successful technique for numerous non-deterministic polynomial-time hard (NP-hard) combinatorial optimization problems such as the traveling salesman problem, the graph coloring problem, the frequency assignment problem, the quadratic assignment problem, feature selection for microarray classification and the vehicle routing problem [17-22]. ACO has the advantages of a positive feedback, and it lends itself to parallel computing, among other advantages.

As defined by Dorigio and Gambardella [13], ACO is comprised of parallel artificial ants that communicate through a probability density function (PDF) that is updated by weights or 'pheromone levels'. In this case, the ACO is an iterative procedure which stops at a pre-defined number of iterations and the weights are determined by the significance of the epistatic interaction of the selected set of SNPs. The probability of selecting locus k at iteration *i *is defined as:(1)

where *τ*_*k*_(*i*) is the amount of pheromones for locus *k *at iteration *i*;  is some form of prior information, which is set to 1 in this study as we treat each locus equally; *α *is the parameter determining the weight given to the pheromones deposited by ants. The ACO is initialized with all loci having an equal level of pheromone *τ*_0_. Using the PDF defined in equation (1), each artificial ant, *m*, will select an SNP set *S*_*m *_of *n *loci from the whole set of genomic SNPs. The epistatic interaction for this SNP set is evaluated by the *χ*^2 ^test. The pheromone level of each locus *k *in *S*_*m *_is then updated, based on the performance of *S*_*m*_, as:(2)

where *ρ *is a constant between 0 and 1 that represents the pheromone evaporation rate; Δ*τ*_*k*_(*i*) is the change in pheromone level for locus *k *at iteration *i*, which equals 0.1 *χ*^2 ^of *S*_*m *_in this study, and is set to zero if locus *k *∉ *S*_*m*_. This process is repeated for all artificial ants.

### AntEpiSeeker algoritms

**Figure 1:**
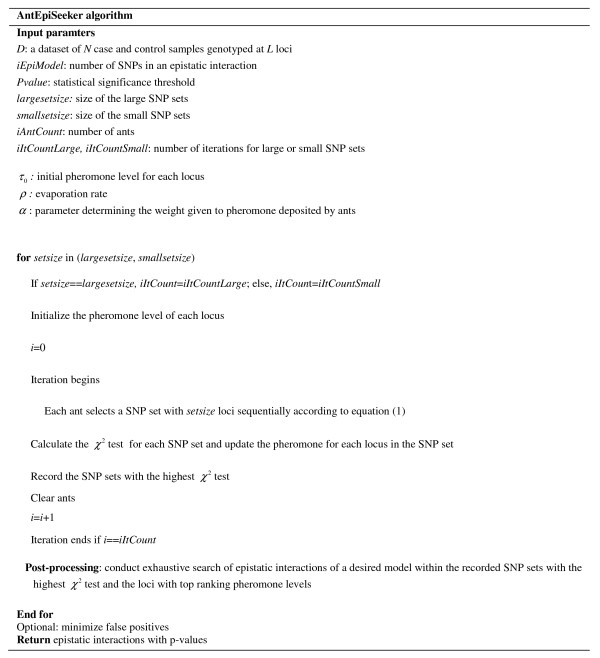
**Pseudocode for AntEpiSeeker**.

In an effort to increase the detection power of the generic ACO as outlined in the previous section, an advanced ACO algorithm called AntEpiSeeker, which employs a two-stage design of ACO, is proposed. The first stage of AntEpiSeeker searches SNP sets of sufficient size (larger than the number of SNPs in a given epistatic interaction) using the above ACO, which results in a pre-defined number of highly suspected SNP sets determined by *χ*^2 ^scores, and another SNP set of a pre-defined size, determined by pheromone levels. The second stage of AntEpiSeeker conducts exhaustive search of epistatic interactions within the highly suspected SNP sets, and within the reduced set of SNPs with top ranking pheromone levels. The use of highly suspected SNP sets (much smaller than the available SNPs in the data) enhances the power of detecting pure epistasis based on greatly reduced computational cost and the SNP set with top ranking pheromone levels is used to detect epistasis among the SNPs with big marginal effects. Additionally, we suggest two rounds of search: 1) using a relatively large size SNP set, which is sensitive to strong signals, and 2) using a relatively small size SNP set, which is sensitive to weak signals. The pseudocode for AntEpiSeeker is shown in Figure 1.

### Minimizing false positives

AntEpiSeeker may report all detected epistatic interactions at a p-value threshold. In addition, AntEpiSeeker incorporates a procedure for minimizing false positives, which can be described as:

1) The set of all detected epistatic interactions is denoted by *EI*_*all *_and another null set, holding the epistatic interactions with minimized false positives, is denoted by *EI*_*m*_.

2) Each reported epistatic interaction *I*_*i *_in *EI*_*all *_is attempted to be added into *EI*_*m *_sequentially. If *I*_*i *_does not have any locus overlapping with those of each epistatic interaction in *EI*_*m*_, *I*_*i *_is added to *EI*_*m*_. Otherwise, assuming that the epistatic interaction *J*_*j *_in *EI*_*m *_has at least one locus overlapping with those of *I*_*i*_, determine if the p-value of *I*_*i *_is smaller than that of *J*_*j*_. If so, *J*_*j *_in *EI*_*m *_is replaced by *I*_*i*_. If not, *I*_*i *_is not reported in *EI*_*m*_.

### The software

The AntEpiseeker package was written in C++. Before compiling, the GNU Scientific Library (GSL) needs to be installed on the user's computer. A separate parameter file named "parameters.txt" specifies the parameters needed to run the program. The SNP data file should be comma-delimited, with the first row specifying the SNP names. All subsequent rows should contain SNP data for each sample. The SNP data should be coded by 0, 1 and 2. The last column indicates the sample status (0 indicates control and 1 indicates case). There are three output files. "AntEpiSeeker.log" records some intermediate results, "results_maximized.txt" reports all detected epistatic interactions, and the user-specified output file shows the epistatic interactions with minimized false positives. The user specified output file includes the locus name, *χ*^2 ^value and p-value. The software and its source code are available for download at http://nce.ads.uga.edu/~romdhane/AntEpiSeeker/index.html.

### Parameter Setting

The parameters needed to run AntEpiseeker include *iAntCount, iItCountLarge, iItCountSmall, α, iTopModel, iTopLoci, ρ, τ*_0_, *largesetsize, smallsetsize, iEpiModel, pvalue, INPFILE, OUTFILE*. The parameter "*iEpiModel*" specifies the number of SNPs in an epistatic interaction. The parameters "*largesetsize*", "*smallsetsize*" must be greater than "*iEpiModel*". For a two-locus interaction model, we suggest *largesetsize *= 6, *smallsetsize *= 3, *iEpiMode *= 2; For a three-locus interaction model, we suggest *largesetsize *= 6, *smallsetsize *= 4, *iEpiModel *= 3. The parameters "*iItCountLarge*", "*iItCountSmall*" should be chosen according to the number of SNPs genotyped in the data (Denoted by *L*). Typically, we suggest *iItCountSmall *≥ 0.1 × *L *and *iItCountLarge *= 0.5 × *iItCountSmall*. *iAntCount *may vary from 500 to 5,000, where larger *iAntCount *should correspond to larger *L*. *ρ *should range from 0.01 to 0.1 for better performance, where smaller *L *should use larger *ρ*. The default parameters in the AntEpiSeeker package, used in our most simulation studies, were an optimal setting balanced between *ρ *and *iAntCount*, which should work well on medium size datasets (2 × 10^3 ^≤ *L *≤ 2 × 10^4^).

## Results

### Power and computational time evaluation on a simulated data set

**Figure 2:**
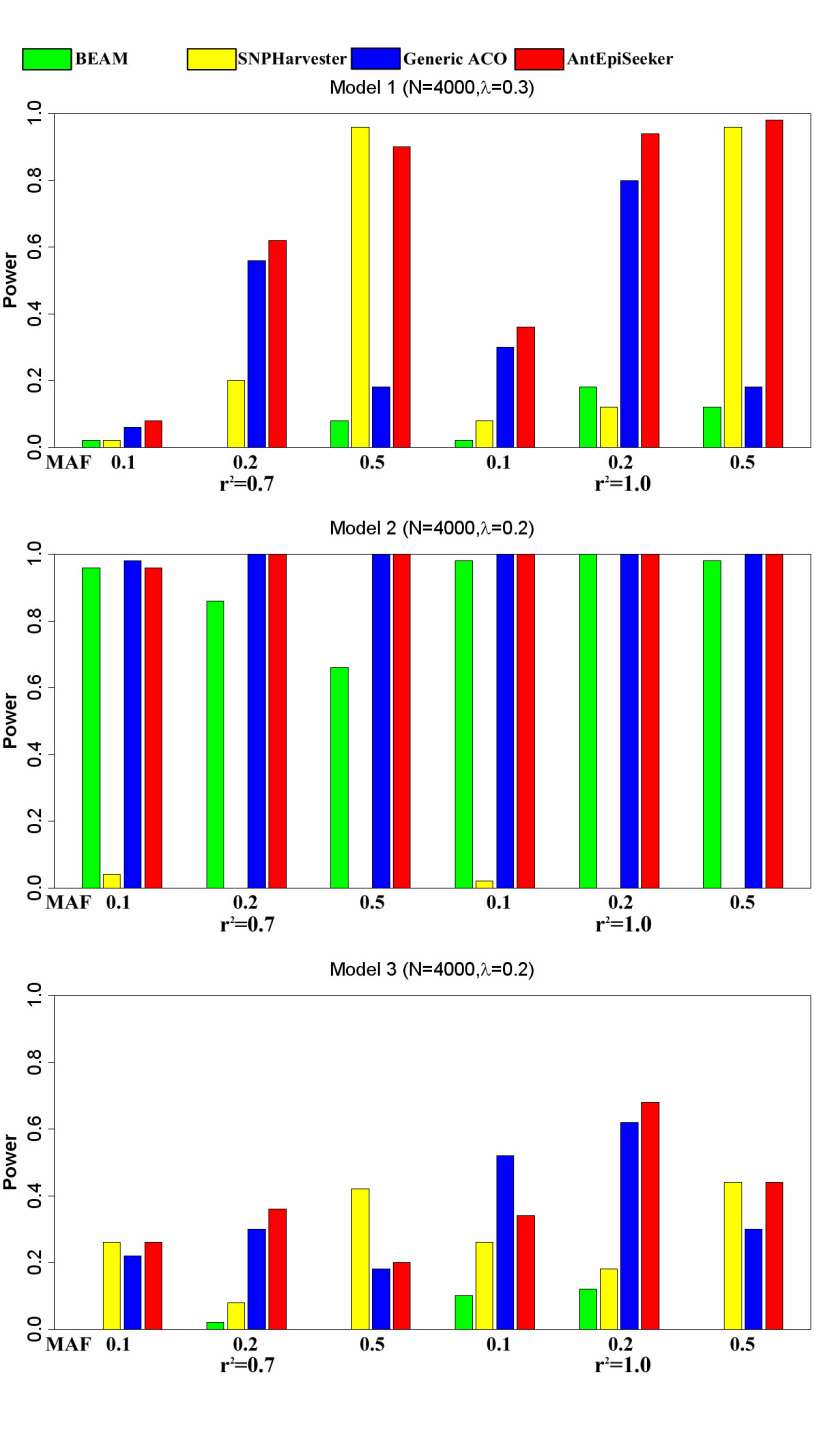
**Power comparison between AntEpiSeeker, SNPHarvester, BEAM and Generic ACO**. The absence of bars indicates zero power.

The performance of AntEpiSeeker was evaluated by comparison with two recent methods, BEAM [11] and SNPHarverster [12], as well as the generic ACO algorithm, using simulated data generated by the simulation program provided in the BEAM package. Note that the generic ACO algorithm does not select SNP sets of bigger size, and thus the parameters "*largesetsize, smallsetsize, iItCountLarge, iItCountSmall*" were not needed for the generic ACO algorithm. The simulation study was conducted following the procedure and parameter settings of many previous studies [11,12,16,23,24]. For each combination of parameter settings, 50 datasets containing 4,000 samples (2,000 cases and 2,000 controls) and 2000 SNPs were simulated. The detection power was calculated as the ratio of the number of successful identifications to the number of datasets at the significance level 0.01 after Bonferroni correction. Data was simulated following three genetic models: 1) additive model, 2) epistatic interactions with multiplicative effects and 3) epistatic interactions with threshold effects, as defined by Marchini et al. [16]. Other parameters for data simulation were the effective size *λ *(a measure of marginal effects as defined by Marchini et al. [16]), linkage disequilibrium between SNPs measured by r^2 ^and minor allele frequencies (MAFs). *λ *was set to 0.3 for Model 1 and 0.2 for Models 2 and 3. For r^2^, two values (0.7 and 1.0) were used for each model. For MAF, three values (0.1, 0.2, and 0.5) were considered. The parameters for BEAM were set as default. The parameter settings for SNPHarvester were: 1 ≤ *k *≤ 5 and *paths *= 50, as suggested by its original simulation study. The parameter settings for AntEpiSeeker were: *largesetsize *= 6, *smallsetsize *= 3, *iItCountLarge *= 150, *iItCountSmall *= 300, *iEpiModel *= 2, *iAntCount *= 1000, *α *= 1, *ρ *= 0.05 and *τ*_0 _= 100 (also available in the software package of AntEpiSeeker). The parameters of the generic ACO algorithm were set as *iAntCount *= 1000, *α *= 1, *ρ *= 0.05, *τ*_0 _= 100, *iItCount *(number of iterations) = 900, *iEpiModel *= 2. The comparison of detection power for AntEpiSeeker, BEAM, SNPHarvester and the generic ACO is presented in Figure 2. The results show that AntEpiSeeker outperforms BEAM in all parameter settings and is superior to SNPHarvester and the generic ACO in most parameter settings.

In addition, AntEpiSeeker is computationally efficient. In the above simulation study, the average running time of AntEpiSeeker, SNPHarvester and BEAM were 27, 54 and 133 minutes respectively, using a Linux system based on Dual Core AMD Opteron(tm) Processor 275.

### False positive rate evaluation on a null simulation

To approximate the false positive rates of AntEpiSeeker, a dataset without any genetic effects was simulated. The dataset contained 2,000 SNPs and 4,000 samples (2000 cases and 2000 controls), whose SNPs were generated independently with MAF uniformly distributed in [0.1, 0.5]. The parameters for running programs were the same as used in the first experiment. At different p-values, the false positive rates of the exhaustive search, BEAM, SNPHarvester and AntEpiSeeker are shown in Table 1. BEAM did not report any false positive, while the false positive rate of SNPHarvester is much higher than exhaustive search. AntEpiSeeker has a false positive rate that is comparable to but no larger than the desired significance level.

**Table 1:** False positive rate of different methods on a null simulation.

	False positive rate				
	
				AntEpiSeeker	
		
P value threshold	Exhaustive search	BEAM	SNPHarvester	Before minimizing false positives	After minimizing false positives
10^-5^	5.5 × 10^-6^	No positives reported	1.4 × 10^-2^	3.5 × 10^-6^	3.0 × 10^-6^
10^-4^	5.3 × 10^-5^	No positives reported	1.6 × 10^-2^	3.0 × 10^-5^	1.1 × 10^-5^
10^-3^	6.9 × 10^-4^	No positives reported	2.0 × 10^-2^	2.9 × 10-4	3.7 × 10^-5^
10^-2^	8.4 × 10^-3^	No positives reported	2.4 × 10^-2^	2.0 × 10^-3^	6.6 × 10^-5^

### Evaluation of AntEpiSeeker on a simulated large scale dataset

To further test the performance of AntEpiseeker, a real data based simulation study was carried out. The dataset consisted of the SNP genotypes on human chromosome 1 from 912 individuals (11 populations) of the International HapMap project (Phase 3) [25]. Loci with missing genotypes or MAF<0.1 were removed, resulting in 73,355 SNP markers for analysis. Because it has no case/control status attached, the data was randomly and equally divided between cases and controls (456 individuals in each group). Additionally, 132 epistatic interactions following the above mentioned 3 models were added to the data with randomly selected causative loci. The p-value threshold was set at 0.0001. The parameters for running programs were the same as used in the first experiment, for a fair comparison. The available software BEAM was not able to handle this dataset. The performances of SNPHarvester, generic ACO and AntEpiSeeker were compared in terms of true positive rates and false discovery rates, as summarized in Table 2. The results suggest that AntEpiSeeker significantly outperforms other methods on this large scale dataset.

**Table 2:** Performance comparison of different methods on a simulated large-scale dataset.

Methods	True positive rate	False discovery rate
SNPHarvester	26.5%	98.6%
Generic ACO	0	100%
AntEpiSeeker	66.7%	97.1%
AntEpiSeeker with minimized false positives	52.3%	18.8%

### Results on WTCCC RA data

AntEpiSeeker was used to perform a large-scale association study on the rheumatoid arthritis(RA) data from the Wellcome Trust Case Control Consortium (WTCCC) [26], which consisted of 332,831 SNP markers and 3,503 individuals (1,504 controls and 1,999 cases). Chromosomes were scanned separately first and then jointly. The parameters for AntEpiSeeker were adjusted according to the general rule presented in the section of parameter setting. Different methods were also compared based on this real dataset. The available software BEAM was not able to handle such a big dataset. Compared with SNPHarvester, AntEpiSeeker identified more SNP markers, which were previously identified as having remarkable marginal effects [26-28], as being involved in epistatic interactions. We summarized these epistatic interactions in Table 3. Other epistatic interactions suggested by AntEpiSeeker were posted on our project web site at http://nce.ads.uga.edu/~romdhane/AntEpiSeeker/index.html. It took about 5 days for AntEpiSeeker to handle the WTCCC RA data, based on Dual Core AMD Opteron(tm) Processor 275, while SNPHarvester took about 2 weeks to handle it, a similar time to the one reported in [12].

**Table 3:** Some epistatic interactions identified by AntEpiSeeker on WTCCC RA data.

Epistatic interactions	Location	Related genes	P value
(rs6457617, rs41443144)	6p21.32-6p25.1	MHC, RP3	<10^-16^
(rs743777, rs9627642)	22q12.3-22q13.31	NR, NR	< 10^-16^
(rs2837960, rs41492246)	21q22.3-21q22.12	NR, NR	< 10^-16^
(rs6920220, rs17165379)	6q23.3-6p25.3	TNFAIP3, NR	< 10^-16^
(rs6457620, rs41454544)	6p21.32-6q21	HLA-DRB1, OSTM1	< 10^-16^
(rs3890745, rs41348151)	1p36.32-1p21.3	MMEL1, NR	< 10^-16^
(rs4810485, rs2748666)	20q13.12-20q11.23	CD40, NR	< 10^-16^

## Conclusion

In this paper, we proposed a novel tool (AntEpiSeeker) for the discovery of epistatic interactions in large scale case-control studies. AntEpiSeeker was assessed through comparison with two recent approaches on both simulated and real datasets. AntEpiSeeker, which adopts a two-stage optimization procedure, is a modified algorithm derived from the generic ACO. To demonstrate the advantages of the two-stage optimization, we also compared the performance of AntEpiSeeker with that of the generic ACO. AntEpiSeeker is a continuous research project and may be upgraded in the future.

## Availability and requirements

**Project name**: AntEpiSeeker

**Project home page**: http://nce.ads.uga.edu/~romdhane/AntEpiSeeker/index.html

**Operating system(s)**: Windows, Linux

**Programming language**: C++

**Other requirements**: GNU Scientific Library (GSL) is needed for recompile

**License**: None for usage

**Any restrictions to use by non-academics**: None

## Competing interests

The authors declare that they have no competing interests.

## Authors' contributions

YW and KR conceived the project. YW developed the software. YW and XL analyzed the data. YW and RR wrote the manuscript. All authors read and approved the final manuscript.
